# Design and Evaluation of Ethyl Cellulose Based Matrix Tablets of Ibuprofen with pH Modulated Release Kinetics

**DOI:** 10.4103/0250-474X.45397

**Published:** 2008

**Authors:** S. Chandran, Laila F. A. Asghar, Neelima Mantha

**Affiliations:** Formulation Development and Pharmacokinetics Laboratory, Pharmacy Group, Birla Institute of Technology and Science, Pilani-333 031, India

**Keywords:** Ibuprofen, ethyl cellulose, matrix tablet, cellulose acetate phthalate

## Abstract

Controlled release preparations have been reported to reduce the gastro irritant and ulcerogenic effects of non steroidal antiinflammatory drugs. In the present study, an attempt was made to develop matrix tablet-based controlled release formulations of ibuprofen, using ethyl cellulose as the rate-controlling polymer. In order to prevent initial release of the drug in the acidic environment of the stomach, cellulose acetate phthalate was incorporated in the matrix in varying amounts. It was found that with increasing the proportion of ethyl cellulose in the matrix, the drug release was extended for 14-16 h. Incorporation of cellulose acetate phthalate in ethyl cellulose matrix provided very low initial release of the drug in the first 2-3 h followed by enhanced release rate in alkaline medium owing to the high solubility of cellulose acetate phthalate at basic pH which led to creation of a porous matrix. It was concluded that combination of cellulose acetate phthalate with ethyl cellulose in the matrix base can be an effective means of developing a controlled release formulation of ibuprofen with very low initial release followed with controlled release up to 14-16 h.

Nonsteroidal antiinflammatory drugs (NSAIDs) are highly effective in the treatment of rheumatoid and osteoarthritis but their long term use is beset with gastrointestinal (GI) toxicity in a large number of cases like ulceration and stricture formation in esophagus, stomach and duodenum leading to severe bleeding, perforation and obstruction^1^–^3^. Ibuprofen, like other drugs of this group, also has a wide spectrum of gastrointestinal side effects ranging from mild dyspepsia to gastric bleeding^4^. Due to its short plasma half-life (1-3 h) and GI toxicity profile, ibuprofen is an ideal candidate for preparing extended or controlled release drug products that can potentially avoid drug release in upper position of the GI tract.

Several matrix based controlled release products of ibuprofen have been reported based on the use of either hydrophilic (HPMC or Carbopol) and/or hydrophobic polymers (EC)^5^–^11^. The reported controlled release formulations of ibuprofen did not involve any attempt to prevent drug release in the upper GI tract. Cellulose acetate phthalate (CAP) is a commonly employed enteric coating polymer in pharmaceutical industry^12^. In combination with cellulose acetate butyrate, CAP has been employed for preparing enteric matrix microspheres by emulsion solvent evaporation technique^13^–^15^. Detailed literature search revealed only one report on the use of CAP in tablet based matrix systems for pH modulated release^16^.

In the present study, it was envisaged to design controlled release formulation of ibuprofen with pH dependent release profile so as to minimize/prevent initial drug release in stomach that will reduce the possible gastro irritant and ulcerogenic effects of the drug. At the same time, there would be no compromise on the biopharmaceutical profile of the drug as ibuprofen is reported to be well - absorbed through out the GI tract^17^.

## MATERIALS AND METHODS

Ibuprofen and cellulose acetate phthalate were obtained as gift samples from Bajaj Organics Ltd, Aurangabad, India and Alkem Laboratories, Mumbai, India, respectively. Ethyl cellulose was purchased from CDH chemicals, New Delhi, India. All other chemicals and reagents used were either of analytical or pharmaceutical grades.

### Analytical method:

Ibuprofen in pure form and designed formulation was analyzed using in-house developed and validated UV spectrophotometric method using Jasco V-570 double beam UV/Vis spectrophotometer (Jasco Corporation, Tokyo, Japan) with built-in Spectra Manager software. The method involved analysis of the drug at 264 nm in 7.4 pH phosphate buffer using 1 cm matched quartz cells.

### Preparation of matrix tablets:

Different matrix embedded formulations of ibuprofen were prepared by wet granulation technique using varying proportion of polymers. Accurately weighed quantities of pre-sieved drug and polymer(s) were mixed thoroughly and granulated with ethyl alcohol. The wet granules were sieved through #20 sieves and the final granules were blended with 1% talc and 0.5% magnesium stearate and compressed using 11 mm punches on 16-station rotary tablet press (Cadmach, Ahmedabad, India). Three batches of tablets were prepared for each formulation. Composition of the prepared matrix embedded tablets of ibuprofen is presented in Table 1.

**TABLE 1:** COMPOSITION AND PHYSICAL PROPERTIES OF VARIOUS DESIGNED FORMULATIONS

Formula	IE1C0	IE1C2	IE1C3	IE2C0	IE2C3	IE2C4	IE4C0
Components# (in mg/tablet)							
Ibuprofen	200	200	200	200	200	200	200
EC	25	25	25	50	50	50	100
CAP	0	50	75	0	75	100	0
Total tab weight	225	275	300	250	325	350	300
Physical Properties							
Drug content (mg / tab)^a^	101±0.5	103±0.7	100±0.5	99±0.8	98±0.5	104±0.5	100±0.1
Weight variation (%)^b^	±3.0	±4.0	±2.0	±2.0	±5.0	±2.0	±3.0
Hardness (Kg/ cm^2^)^c^	5.5±0.1	5.2±0.4	6.1±0.3	6.7±0.2	5.2±0.1	5.3±0.2	6.0±0.2
Friability (%)^d^			< 0.5%				

# Also contains 1% w/w talc and 0.5% w/w magnesium stearate as formulation additives.

a % w/w of the drug content.

b ±max % variation from the mean value.

c mean of 10 tablets with SD.

d mean of 20 tablets.

### Physicochemical characterization of tablets:

Drug content of the manufactured tablets of each batch was determined by weighing and finely grinding twenty tablets from each batch. Aliquot of this powder equivalent to 100 mg of ibuprofen was accurately weighed, suitably extracted in 50 ml of 0.1 N NaOH. The resulting solution was filtered, suitably diluted using phosphate buffer pH 7.4 and analyzed by UV spectrophotometric method at 264 nm. The weight variation was evaluated on 10 tablets using an electronic balance (Mettler Toledo, Mettler, Griefensee, Switzerland). Tablet hardness was determined for 10 tablets using a Monsanto (standard type) tablet hardness tester. Friability was determined taking 20 tablets in a Campbell Electronic Friabilator for 4 min at 25 rpm. The physicochemical properties of designed tablets are shown in Table 1.

### *In vitro* release rate studies:

**Fig. 1:**
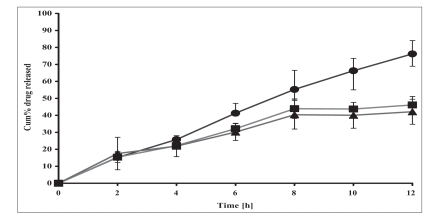
Release profile of ibuprofen from batches with varying EC content Release profile of ibuprofen from batches with varying EC content, IE1C0- 12.5% (–●–), IE2C0- 25%(–■–), and IE3C0- 50%(–▲–) w/w of drug. Each data point represents mean±SD of n = 6.

**Fig. 2:**
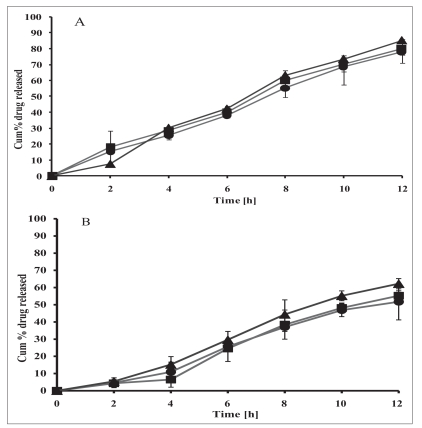
Release profile of ibuprofen from formulations Release profile of ibuprofen from formulations containing A: 12.5% w/w EC with varying CAP content, IE1C0- 0%(–●–), IE1C2- 25%(–■–), IE1C3- 37.5%(–▲–) w/w of drug and B) containing 25% w/w EC with varying CAP content IE2C0- 0%(–●–), IE2C3- 37.5%(–■–), IE2C4- 50%(–▲–) w/w of drug. Each data point represents mean ± SD (*n* = 6).

*In vitro* drug release rate studies were carried out using USP Type II dissolution apparatus (Paddle method) at 37±1° and 75 rpm. Since ibuprofen is practically insoluble in acidic pH, the dissolution media composition maintained was 500 ml of distilled water for first two hours followed with addition of 200 ml of concentrated phosphate buffer to raise the pH to 7.4 for the remaining period of the study. At predetermined time intervals, 10 ml sample was withdrawn and replaced with fresh medium. Samples were filtered through Whatman filter paper (No- 41) and analyzed after suitable dilution. All dissolution studies were carried out in duplicate and repeated at least thrice. The results are shown in figs. 1, 2.

### Effect of simulated GI fluid pH (without enzymes) and surfactants on the release profile:

**Fig. 3:**
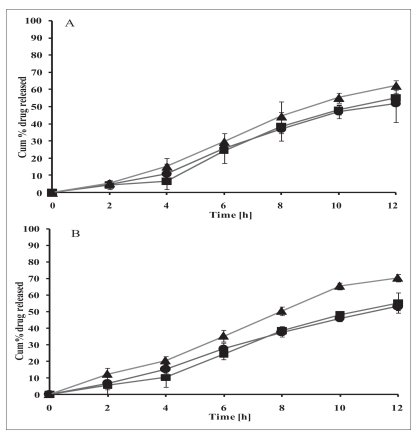
Release profile of ibuprofen from the formulations IE1C3 and IE2C4 Release profile of ibuprofen from the formulations A. IE1C3 and B. IE2C4 showing the effect of simulated GI pH (without enzymes) without surfactant (–■–), with 1% v/v Tween 80 (–●–) and with 0.5%w/v SLS (–▲–). Each data point represents mean ± SD (*n* = 6).

The effect of simulated GI fluid pH (without enzymes) on the release characteristics of selected formulations (IE1C3 and IE2C4) was studied. For this purpose, the following protocol was employed. The release profile was studied in a medium of simulated GI pH by starting with a tablet in 350 ml of 0.2 M HCl for 2 h. Then, 190 ml phosphate buffer (17.1 g of KH_2_ PO_4_.12H_2_ O) and 60 ml of 0.5 M NaOH in distilled water) was added to raise the pH of the media to 4.5 and total dissolution volume to 600 ml. At the end of 4 h, pH was raised to 7.4 by adding 300 ml phosphate buffer (2.18 g of KH_2_ PO_4_ and 1.46 g of NaOH prepared in distilled water) and the dissolution was carried out in this medium (final total volume 900 ml) till the end of study. At predetermined time intervals, a 10 ml sample was withdrawn and replaced with fresh dissolution media up to 12 h. After appropriate dilutions, the samples were analyzed. The results are presented in fig. 3

### Determination of release kinetics:

The release mechanism and the release rate constants (% h^−1^) were elucidated by the method given by simple power equation^18^. This was done by fitting the dissolution data in Eqn. 1, M_t_ /M_∞_ = Kt^n^ (1), where, M_t_ /M_∞_ is fraction of drug released at any time ‘t’; K is release rate constant incorporating the structural and geometric characteristics of the tablets; n is the diffusional exponent, indicative of the release mechanism. [The value of n for a cylinder is < 0.45 for Fickian release, > 0.45 and < 0.89 for non- Fickian release, 0.89 for case II release and > 0.89 for super case II release]. The values of K, n, r (correlation coefficient) and time required for 10%, 50% and 90% of drug release (t_10%,_ t_50%_ and t_90%_ respectively) and as obtained from the dissolution data of designed formulations are given in Table 2. The release kinetics parameters for formulations studied in simulated GI pH (without enzymes) with and without surfactant are listed in Table 3.

**TABLE 2:** RELEASE KINETICS PARAMETERS FOR DESIGNED FORMULATIONS

Parameters	IE1C0	IE1C2	IE1C3	IE2C0	IE2C3	IE2C4	IE4C0
r^a^	0.9973	0.9926	0.9814	0.9830	0.9780	0.9729	0.9754
K^b^	0.0770	0.0941	0.0871	0.0658	0.0883	0.0905	0.0365
n^c^	0.72	0.85	0.87	0.66	1.38	1.32	0.54
t_10%_^d^	1.1	1.1	2.4	1.3	2.4	2.6	1.3
t_50%_^e^	7.5	6.9	7.9	13.5	7.6	7.9	15.0
t_90%_^f^	14.9	15.2	14.5	27.1	14.2	14.4	29.1

a Correlation coefficient.

b Release rate constant.

c Diffusional exponent indicative of the release mechanism.

d Time for 10% of the drug release (in h).

e Time for 50% of the drug release (in h).

f Time for 90% of the drug release (in h).

**TABLE 3:** RELEASE KINETICS PARAMETERS FOR IE1C3 AND IE2C4 IN SIMULATED GI FLUID pH (WITHOUT ENZYMES) WITH AND WITHOUT SURFACTANT

Parameters	IE1C3			IE2C4		
		
	Without surfactant	Tween 80 (1% v/v)	SLS (0.5% w/v)	Without surfactant	Tween 80 (1% v/v)	SLS (0.5% w/v)
r^a^	0.9685	0.9923	0.9933	0.9891	0.9802	0.9711
K^b^	0.0119	0.0174	0.0224	0.0192	0.0127	0.0157
n^c^	1.50	1.42	1.31	1.38	1.32	1.41
t_10%_^d^	3.1	3.4	2.9	3.3	3.7	3.0
t_50%_^e^	10.5	10.6	9.4	10.5	11.1	7.8
t_90%_^f^	15.3	16.1	14.4	16.1	17.1	14.7

a Correlation coefficient.

b Release rate constant.

c Diffusional exponent indicative of the release mechanism.

d Time for 10% of the drug release (in h).

e Time for 50% of the drug release (in h).

f Time for 90% of the drug release (in h).

### Batch reproducibility and stability on storage:

Three batches of each formulation were prepared and their respective dissolution rates were evaluated under the same conditions. The best formulation of each type was studied after 6 mo and 1 y for the effect of storage in ambient conditions on the stability and release profiles of drug from the different formulations respectively. The tablets were sealed in airtight cellophane packets and were stored in ambient conditions (temperature- 25° and relative humidity- 65%). The *In vitro* release profile for each was studied as per the specification enlisted in previous sections and compared with its initial release profile.

### Data analysis:

The difference in the release data for the different formulations was compared using paired t-test for means and one-way analysis of variance (ANOVA) at 5% level of significance using Microsoft Office 2003, Excel package.

## RESULTS AND DISCUSSION

Physical appearance, hardness, friability, weight variation and drug content uniformity of different tablet formulations were found to be satisfactory (Table 1). The manufactured tablets showed low weight variation and high degree of drug content uniformity.

Ibuprofen, a weak propionic acid derivative with a pKa of 5.5 is practically insoluble in simulated gastric fluid. Therefore, dissolution studies were carried out in distilled water for the first two hours followed by phosphate buffer pH (7.4) for the remaining period of study^19^. This medium was considered as most suitable as the drug was freely soluble at this pH and it also mimics the alkaline environment of small intestine. The selection of wet granulation technique for matrix tablet preparation was based on previously reported study which suggested that wet granulation results in harder tablets with lower matrix porosity that give very low release rates when compared to direct compression^20^. In our study, the use of ethyl alcohol as granulating agent was based on the partial solubility of EC in this granulating solvent which resulted in providing the necessary adhesion between the various matrix components and precluded the use of a separate binder.

The release profiles of matrix tablets of ibuprofen containing varying proportions of EC (12.5%, 25% and 50% w/w of drug), i.e., IE1C0, IE2C0 and IE4C0 respectively are shown in fig. 1. The t_10%_ values varied between 1.1 to 1.3 h for these formulations indicating that the initial release kinetics of the drug from the different matrices was practically the same irrespective of the different levels of EC incorporated in the matrix base (Table 2). However, the calculated t_50%_ (IE1C0- 7.5 h, IE2C0- 13.5 h and IE4C0- 15 h) and the t_90%_ (IE1C0- 14.9 h, IE2C0- 27.1 h and IE4C0- 29.1 h) values for these three formulations showed that there was significant retardation in the drug release rate when the proportion of EC was doubled from 12.5% w/w to 25% w/w of drug. It has been previously reported that high levels of EC reduce drug release rates on account of formation of a strong matrix with reduced porosity. This increases diffusional path length leading to reduced water penetration through the micropores resulting in slower drug release^20^. However, on subsequent increase to 50% w/w of drug, there was no appreciable decrease in the release rate and extension in duration of release. This indicated that a tight non-porous matrix had been formed in the former case and addition of more polymer could not modify the matrix character any further. Hence, further studies were continued with formulations comprising of EC at either 12.5% w/w or 25% w/w of drug.

In a previously reported study, matrix tablets of ibuprofen and EC prepared by direct compression have shown the predominance of diffusion (Fickian) as the mechanism of release^11^. However, in our case, the release mechanism followed non-Fickian release pattern (n value between 0.45 and 0.89), which may result from swelling and relaxation of EC in the matrix in the dissolution medium. This might be due to a possible change in the polymer character due to wet granulation with ethyl alcohol (Table 2).

As the presence of only EC in the matrix would not give the desired release profile of low initial drug release followed by increased release rate, CAP was included in the matrix. It was expected that presence of CAP would confer pH modulated release characteristics with very low drug release in acidic environment of the upper GI tract (stomach and initial duodenum) followed by higher release rate in the alkaline pH of small intestine on account of formation of a porous matrix due to dissolution of CAP.

The release profiles of ibuprofen matrix tablets (IE1C2 and IE1C3) containing fixed proportion of EC (12.5% w/w of drug) with CAP at 25% w/w and 37.5% w/w of drug respectively are compared with the corresponding matrix formulation of drug containing EC alone (IE1C0; 12.5% w/w of drug) (fig. 2a). The calculated t_10%_ values (Table 2) for the formulations IE1C0 (1.1 h) and IE1C2 (1.1 h) indicated practically no difference in the initial drug release. However, incorporation of CAP in matrix at 37.5% w/w of drug (IE1C3) gave a comparatively lower initial release in first 2 h with t_10%_ of 2.4 h. The calculated t_50%_ (IE1C0- 7.5 h, IE1C2- 6.9 h and IE1C3- 7.9 h) and the t_90%_ (IE1C0- 14.9 h, IE1C2- 15.2 h and IE1C3- 14.5 h) for the three formulations were not found to be significantly different from each other indicating only a marginal change in release rate post 2 h. This indicated that CAP was not present in sufficient proportion to influence drug release pattern from the matrix. The release mechanism was again found to be of non-Fickian type (n is > 0.45 and < 0.89) for all the three formulations indicating anomalous nature of release (Table 2).

Alternately, when the release profiles of ibuprofen matrix tablets containing fixed proportion of EC (IE2C0; 25% w/w of drug) alone or with varying proportions of CAP (IE2C3; 37.5% w/w and IE2C4; 50% w/w of drug) were compared, a significant difference was observed in the release rate and nature of release between these matrices (fig. 2b). The calculated t_10%_ values for the three formulations (IE2C0- 1.3 h, IE2C3- 2.4 h and IE2C4- 2.6 h) indicated good retardation in the initial release of the drug in distilled water from the two matrices containing CAP as compared to the one with EC alone (Table 2). Further, as expected, the rate of release of drug from these matrices was found to increase when the media was changed to phosphate buffer pH 7.4 with the calculated t_50%_ values obtained as 13.5 h (IE2C0), 7.6 h (IE2C3) and 7.9 h (IE2C4) and the t_90%_ values as 27.1 h (IE2C0), 14.2 h (IE2C3) and 14.4 h (IE2C4). The lower t_50%_ and t_90%_ values in case of CAP containing EC matrix tablets in comparison to tablet containing EC alone can be attributed to increased erosion of CAP containing tablets at pH 7.4. Also, since 90% drug release was attained in about 14-15 h it can be expected that drug release would be complete within the residence time of the dosage form in the GI tract. These results were similar to a previously reported study wherein a binary mixture of EC and CAP were used to prepare microspheres of acetyl salicylic acid and the release was found to depend on pH of dissolution medium^21^. The reason was attributed to the preferential solubility of the drug as well as CAP above pH 6.0. This is due to the formation of a porous and eroded matrix upon dissolution of CAP at higher pH. The same study also reported that unlike pure EC based matrices that give simple Fickian type of drug release, increased erosion and tablet disintegration in the presence of CAP shifts release mechanism to erosion type. In the present case, the release mechanism was found to shift from non-Fickian type with n value >0.45 and < 0.89 for IE2C0 to n > 1.3 for IE2C3 and IE2C4 indicating super case II type release. Moreover, a plot of cumulative % release vs. time (post 2^nd^ h of release) yielded a good correlation coefficient (r = 0.9849 and 0.9975) for IE2C3 and IE2C4, respectively, confirming the rate kinetics to be zero-order after the first two hours. This will ensure predictable controlled release behavior of the drug on its passage through the GI tract. As formulations IE1C3 and IE2C4 seemed to be quite promising for controlled drug release with a good initial lag time, they were taken for further studies in simulated GI fluid pH (without enzymes).

The development of a dissolution medium for poorly soluble drugs in which dissolution of the drug becomes the rate limiting step has always been a challenge. Previous studies have shown that the incorporation of synthetic surfactants in dissolution medium is a good means for enhancing the dissolution rate of such drugs^22^. Surfactants like Tween 80 and sodium lauryl sulfate mimic the naturally occurring surfactants and micellar systems present in the human gastrointestinal tract. When present in optimum levels, these surfactants act by enhancing the wettability of a poorly soluble drug and when present above critical micelle concentration, they act by increasing its solubility in dissolution medium^23^. In another study, it has been shown that the addition of Tween 80 did not enhance but actually impeded the dissolution of a poorly soluble drug^24^.

Since ibuprofen has poor solubility at pH 1.2 and 4.5, it was decided to incorporate surfactants in the dissolution media in order to rule out the possibility of dissolution media pH controlling the drug release kinetics from the formulations as against the polymeric matrix base of the formulation. The surfactants used in the simulated GI fluid pH (without enzymes) were either 1% v/v Tween 80 or 0.5% w/v sodium lauryl sulphate (SLS). The cumulative percentage drug release from the two formulations IE1C3 and IE2C4 in simulated GI fluid pH (without enzymes) with or without surfactant are shown in fig. 3a and 3b respectively. The corresponding release kinetics data are presented in Table 3. The initial retardation of release (in the first four hours) was more in simulated GI fluid pH (without enzymes) without surfactants (fig. 3a and 3b) as compared to phosphate buffer pH 7.4 for IE1C3 (fig. 2a) and IE2C4 (fig. 2b). This could be due to the presence of CAP which does not become soluble until pH 6.0.

As can be seen in fig. 3a (IE1C3) the addition of 1% v/v Tween 80 did not significantly increase the release rate of the drug from the formulation in the simulated GI fluid pH (without enzymes). A one-way ANOVA test at 5% level performed between mean cumulative percentage drug release values in simulated GI fluid pH (without enzymes) with and without 1% v/v Tween 80 for both formulations did not show any difference as the calculated F-value (F_Calc_) was found to be less than the critical F-value (F_Crit_). However, a statistically significant difference was obtained at 5% level of significance when one way ANOVA or paired t-test for means was employed for release data obtained in 0.5% w/v SLS and without it.

Since incorporation of 0.5% w/v SLS only marginally increased the dissolution rate of the drug in simulated GI fluid pH (without enzymes) it can be concluded that low solubility of drug and hence saturation solubility of the drug in dissolution media was not the reason for controlled release kinetics of ibuprofen from the designed formulations. On the contrary the controlled release profile of the designed tablets was formulation composition mediated.

In the case of IE2C4, the calculated t_10%_ values in simulated GI fluid pH (without enzymes) containing 0.5% w/v SLS and without SLS was 3.0 h and 3.3 h respectively with corresponding t_90%_ of 14.7 h and 16.1 h indicating marginal difference in the release profile in the presence or absence of SLS in the dissolution media (Table 3). Similarly the calculated t_10%_ and t_90%_ values for IE1C3 as given in Table 3 showed marginal effect of presence or absence of SLS in the dissolution media in the drug release kinetics. For both the formulations effect of 1% v/v Tween 80 was not observed as the release profile was similar to that obtained for without Tween 80. From the present study, it was concluded that while addition of 1% v/v Tween 80 in dissolution media did not affect the release behavior of ibuprofen formulations, presence of 0.5% SLS did show a statistically significant difference in the drug release pattern. Therefore, use of SLS or other surfactants can be a useful means in overcoming dissolution limited release of controlled formulations of poorly soluble drugs.

No significant difference was observed in the release profile of different batches of each matrix formulation, indicating that the manufacturing process employed was reliable and reproducible. Also, the release kinetics remained unaltered up to one year of storage and there were no changes in the tablet characteristics, suggesting that ibuprofen was stable in EC matrices.

In conclusion, matrix embedding technique using EC as the retardant has successfully extended the release of ibuprofen from its tablet formulations. In the present case, we found that the incorporation of CAP in the matrix not only helped to provide good initial retardation in the release but also helps to enhance the overall release rate of the drug after a suitable lag time. The manufacturing method employed is simple and easily adaptable in the conventional tablet-manufacturing units.
